# 

**Published:** 1913-01-25

**Authors:** 


### Answers to Correspondents.

Enquirer asks : In the event of any member of the nursing or domestic staff being taken ill, and staying in the hospital for treatment, can we legally deduct the cost of same from the amount allowed—*i.e.*, from her salary? Who will be responsible for the payment of medical man?

1. The legality of any such deduction is a matter that could only be decided with full knowledge of all the facts, and particular attention would have to be given to the terms of the contracts (if any) under which service is being rendered. In the absence of express stipulation to the contrary in the contracts (if any) it would seem reasonable to make a charge for the treatment given, and it would seem to be necessary, if the legality of such charge were disputed by the nurse or servant, for her to show, from sufficient evidence, that the custom of providing free treatment in sickness was generally recognised, and that it was an inducement to her in seeking the employment. The sickness benefit as such cannot legally be charged or claimed by the hospital, and, in fact, the position from a strictly legal point of view is not altered by the National Insurance Act. We know, however, as a matter of fact, that large London firms which have in the past paid their employees full wages during sickness have adopted the plan of paying the full wages, less the amount receivable as sickness benefit. If this is justified it would certainly appear to be feasible to make a deduction from the salary when treatment is given to a nurse or servant in the institution in which she is normally employed.

2. The nurse or servant falling ill is entitled to free attendance and treatment from the doctor on the panel whom she has chosen, or the doctor with whom she has made her own arrangements with the sanction of the Insurance Committee. If she is treated by anyone else the arrangement is private, and not in the scope of the Act.

### Books Received.

J. WILLING, LTD.: "Willing's Press Guide, 1913."

J. NISBET AND CO.: "Nisbet's Medical Directory, 1913."

"THE PRESCRIBER" OFFICE: "The Prescriber," Vol. VI., 1912.

BAILLIÈRE, TINDALL AND COX: "Midwifery Made Easy," by M. L. Skinner.

UNIVERSITY OF LONDON PRESS: "Diseases of the Skin," by Willmott Evans; "Minor Surgery," by L. A. Bidwell.

### BEQUESTS TO BRISTOL HOSPITALS.

BY the death of Mrs. Annie Ethel Perrin, widow of a tobacco manufacturer of Bristol, some £16,000 now becomes available for charitable purposes. This sum is to be divided into seventeen parts, of which one part goes to each of the following :—The Swansea Hospital; the Royal Ear Hospital, Soho Square, W.; and the Royal Infirmary, the General Hospital, the Dispensary, the Eye Hospital, the Hospital for Women, the Deaf and Dumb Institution, and the Blind Asylum, Bristol.

THE COURT'S SOAP.

Messrs. Edward Cook and Co., Ltd., the Soap Specialists, of Bow, E., have been granted a Royal Warrant of Appointment.

### Buildings Contemplated.

Dartmouth.—The Corporation Works Committee has considered plans and estimates submitted by the surveyor for a new isolation hospital at Jawbones, and recommend that the Council confer with the Port Sanitary Authority as to the advisability of having a combined institution for the port and the town before proceeding further.

Dewsbury.—The Dewsbury Joint Board has decided to take up a loan of £2,090 in connection with a new diphtheria block and verandahs at the Fever Hospital.

Dublin.—Mr. Thos. F. McNamara, C.E., 192 Great Brunswick Street, Dublin, is architect to the Dublin Joint Hospital Board. Tenders are invited for works, including new male wing, new wing for acute cases, entrance lodge, gateway, and new approach road at the Sanatorium, Crooksling, Brittas, Co. Dublin. Sureties are required and the contract contains a fair-wages clause.

Falkirk.—The Public Health Committee, Falkirk, has decided to spend £4,000 to £5,000 on a new sanatorium. Plans are to be forwarded to the Local Government Board for approval.

Festiniog.—The Guardians' Committee is to confer with the Asylums Board Committee regarding a proposed conversion of the workhouse into a branch asylum.

Forfarshire.—The Forfarshire District Committee has appointed a representative to confer with the Dundee Town Council on the advisability of erecting a county sanatorium.

Rainhill (Lancs.).—Mr. R. Wainwright, George Street, St. Helens, is architect for alterations and additions at Lancashire County Asylum, Rainhill. Tenders will presently be considered.

Sandon.—The Public Health Committee of Essex County Council propose to erect a fifty-bed sanatorium at Sandon.

Thornhill.—Derbyshire County Council has authorised the Asylums Committee to expend up to £1,000 for preparation of plans and other preliminaries in connection with the proposed asylum. Messrs. Everard, Son, and Pick, of Leicester, are architects for the scheme.

Walker.—A new operating theatre has been erected at Walker Hospital from a design by Mr. E. F. W. Liddle, of Newcastle.

Wellington.—A committee of Salop County Council has held an inquiry regarding the establishment of an isolation hospital to serve the following districts:—Dawley Urban, Newport Urban and Rural, Oakengates Urban, Shifnal Rural, Wellington Urban and Rural, and a portion of Wenlock Borough.

Wimbledon.—The Local Government Board has fixed a date for inquiry into the Town Council's application for sanction to a £4,000 loan to cover a pavilion for scarlet-fever patients at the Gap Road Isolation Hospital.

### Appointments.

Mr. J. C. Wootton has been promoted from third to second assistant medical officer at Cane Hill Mental Hospital.

Mr. H. M. Prins has resigned his appointment as second assistant medical officer at Colney Hatch Mental Hospital, as he is proceeding to Australia.

The following appointments have been made at the Edinburgh Royal Infirmary: Non-resident house surgeons—James Gossip, M.B., Ch.B., to Dr. George Mackay; and James Maxwell, M.B., Ch.B., to Dr. J. Malcolm Farquharson. Clinical assistants—John W. Gray, M.B., Ch.B., to Dr. Malcolm Farquharson; and A. G. Ritchie, B.Sc., M.B., Ch.B., to Dr. Edwin Bramwell, medical waiting-room.

### Gifts and Bequests.

Mr. John Woodhouse, of Sheffield, retired grocer, who left estate valued at £60,287 gross, has bequeathed £100 each to four Sheffield hospitals.

Mrs. Margaret Bulley, widow of the late President of Magdalen College, Oxford, has left 6d. per week for life to Annie Trinder and £100 to the Fairford Cottage Hospital.

Mr. Austen Chamberlain has received £2,004 from Messrs. F. and A. Swanzy, Limited, being their collection on behalf of the extension of the London School of Tropical Medicine.

Mr. George Enoch Grayson, F.R.I.B.A., of Rock Ferry, Cheshire, designer of numerous churches and public buildings in Liverpool, has bequeathed £200 to the Birkenhead Borough Hospital.

The Queen's Hospital for Children, Hackney Road, has received a cheque for £331 7s. from the Editor of *Little Folks,* towards the funds of its seaside branch, the Little Folks' Home at Bexhill.

Miss Frances Rainer Harris, of Reading, has left, subject to the life interest of her sister, £1,000 each to the Helena Nursing Home, Reading, and to the Royal Asylum of St. Anne's Society, Redhill.

The Royal Children's Subscriptions to the King's Fund.—The Prince of Wales has sent £100 and Prince Albert, Prince Henry, Prince George, Prince John, and Princess Mary £1 1s. each to King Edward's Hospital Fund for London.

The Leicester Royal Infirmary has received intimation of a legacy of £100 under the will of the late Mr. Joseph Gilbert. It has also received from the estate of Mrs. Ellen Dyke Frost £985, the balance realised on winding-up the estate, making in all a total of £2,985.

